# An Objective Screening Method for Major Depressive Disorder Using Logistic Regression Analysis of Heart Rate Variability Data Obtained in a Mental Task Paradigm

**DOI:** 10.3389/fpsyt.2016.00180

**Published:** 2016-11-04

**Authors:** Guanghao Sun, Toshikazu Shinba, Tetsuo Kirimoto, Takemi Matsui

**Affiliations:** ^1^Graduate School of Informatics and Engineering, The University of Electro-Communications, Tokyo, Japan; ^2^Department of Psychiatry, Shizuoka Saiseikai General Hospital, Shizuoka, Japan; ^3^Graduate School of System Design, Tokyo Metropolitan University, Tokyo, Japan

**Keywords:** heart rate variability, major depressive disorder, self-rating depression scale, logistic regression analysis, autonomic activity

## Abstract

**Background and objectives:**

Heart rate variability (HRV) has been intensively studied as a promising biological marker of major depressive disorder (MDD). Our previous study confirmed that autonomic activity and reactivity in depression revealed by HRV during rest and mental task (MT) conditions can be used as diagnostic measures and in clinical evaluation. In this study, logistic regression analysis (LRA) was utilized for the classification and prediction of MDD based on HRV data obtained in an MT paradigm.

**Methods:**

Power spectral analysis of HRV on R–R intervals before, during, and after an MT (random number generation) was performed in 44 drug-naïve patients with MDD and 47 healthy control subjects at Department of Psychiatry in Shizuoka Saiseikai General Hospital. Logit scores of LRA determined by HRV indices and heart rates discriminated patients with MDD from healthy subjects. The high frequency (HF) component of HRV and the ratio of the low frequency (LF) component to the HF component (LF/HF) correspond to parasympathetic and sympathovagal balance, respectively.

**Results:**

The LRA achieved a sensitivity and specificity of 80.0 and 79.0%, respectively, at an optimum cutoff logit score (0.28). Misclassifications occurred only when the logit score was close to the cutoff score. Logit scores also correlated significantly with subjective self-rating depression scale scores (*p* < 0.05).

**Conclusion:**

HRV indices recorded during a MT may be an objective tool for screening patients with MDD in psychiatric practice. The proposed method appears promising for not only objective and rapid MDD screening but also evaluation of its severity.

## Introduction

Major depressive disorder (MDD) is a critical public health concern, with an estimated 350 million people having this disease worldwide (1). Therefore, rapid and accurate detection of patients with MDD during the early stages can facilitate the decision-making process of psychiatric clinicians in the mental health treatment of patients. Unlike other areas of medicine, objective diagnostic biomarkers for psychiatric diseases are not readily available (2, 3). Psychiatric clinicians diagnose outpatients subjectively based on clinical interview and diagnostic criteria. Thus, the diagnosis of MDD tends to depend on the knowledge and experience of the clinician (4). In this study, we propose a novel, non-invasive, objective, and physiological method for screening outpatients with MDD that uses quantitative estimates of altered autonomic nervous system activity and reactivity based on heart rate variability (HRV) indices *via* logistic regression analysis (LRA).

Heart rate variability, which refers to beat-to-beat alterations in the heart rate (HR), can be used to assess autonomic nervous system function (5). Low frequency (LF: 0.04–0.15 Hz), high frequency (HF: 0.15–0.4 Hz), and LF/HF HRV indices provide quantitative estimates of sympathetic and parasympathetic activities (6). Changes in HRV have been found in various disorders presenting both somatic and psychological symptoms, including eating disorder, pain, anxiety, stress disorder, and depression (7–9). Although HRV changes appear non-specific, frequent comorbidity of these disorders may underlie the prevalence. Recent studies have shown a strong association between MDD and HRV abnormalities (10–12). A recent review investigated the impact of depression on HRV indices in patients with MDD without cardiovascular disease (13). High HR and LF/HF and low HF among patients with MDD reflect a baseline shift of autonomic balance toward sympathetic activation (14–16).

In addition to altered autonomic baseline activity, we further assessed the reactivity of HRV measurements during rest and mental task (MT) conditions in patients with MDD and healthy control subjects (17, 18). We found that both HRV at rest and its response to MT conditions were different in patients with MDD. Specifically, HF, LF/HF, and HR were less reactive in patients with MDD compared with healthy subjects during the MT condition. Differences in the reactive of HRV indices during rest following the MT condition were also observed. These multiple autonomic alterations in MDD are possibly related to disturbances in arousal control. These findings suggest that HRV indices may be a useful screening tool for MDD in clinical practice. One such method involves the use of HF, LF/HF, and HR before, during, and after an MT in LRA.

In the present study, a prediction model for binary classification of multidimensional data (i.e., HF, LF/HF, and HR indices) was constructed using multiple LRAs. LRA, which is a probabilistic statistical classification model, is a simple but powerful linear algorithm for analyzing multidimensional data and generating predictions of clinical results (19). The aim of this study was to evaluate the efficacy of a proposed screening method for risk stratification of patients with MDD in clinical settings.

## Materials and Methods

### Participants

We tested this method with clinical data from 44 patients, thereby performing a qualitative assessment of the proposed screening method based on LRA for risk stratification of patients with MDD. The patients (43 ± 12 years old; 23 men and 21 women) from Shizuoka Saiseikai General Hospital were drug-naïve patients and had a diagnosis of MDD according to the Diagnostic and Statistical Manual of Mental Disorders, 4th edition (DSM-IV) criteria published by the American Psychiatric Association. Forty-seven age- and sex-matched healthy control subjects (41 ± 12 years old; 21 men and 26 women) who had never been diagnosed with cardiac, neurological, or psychiatric disorders were also included. There was no difference in the proportion of tobacco users between patients and healthy subjects. Participants were instructed not to consume alcohol or coffee for 24 h prior to the study and not to consume tobacco on the day of the study. The Zung’s self-rating depression scale (SDS) score was used to assess the severity of symptomatology for both groups (20). This study was approved by the Ethics Committee of Shizuoka Saiseikai General Hospital. All subjects provided written informed consent.

### Study Protocol

**Figure 1:**
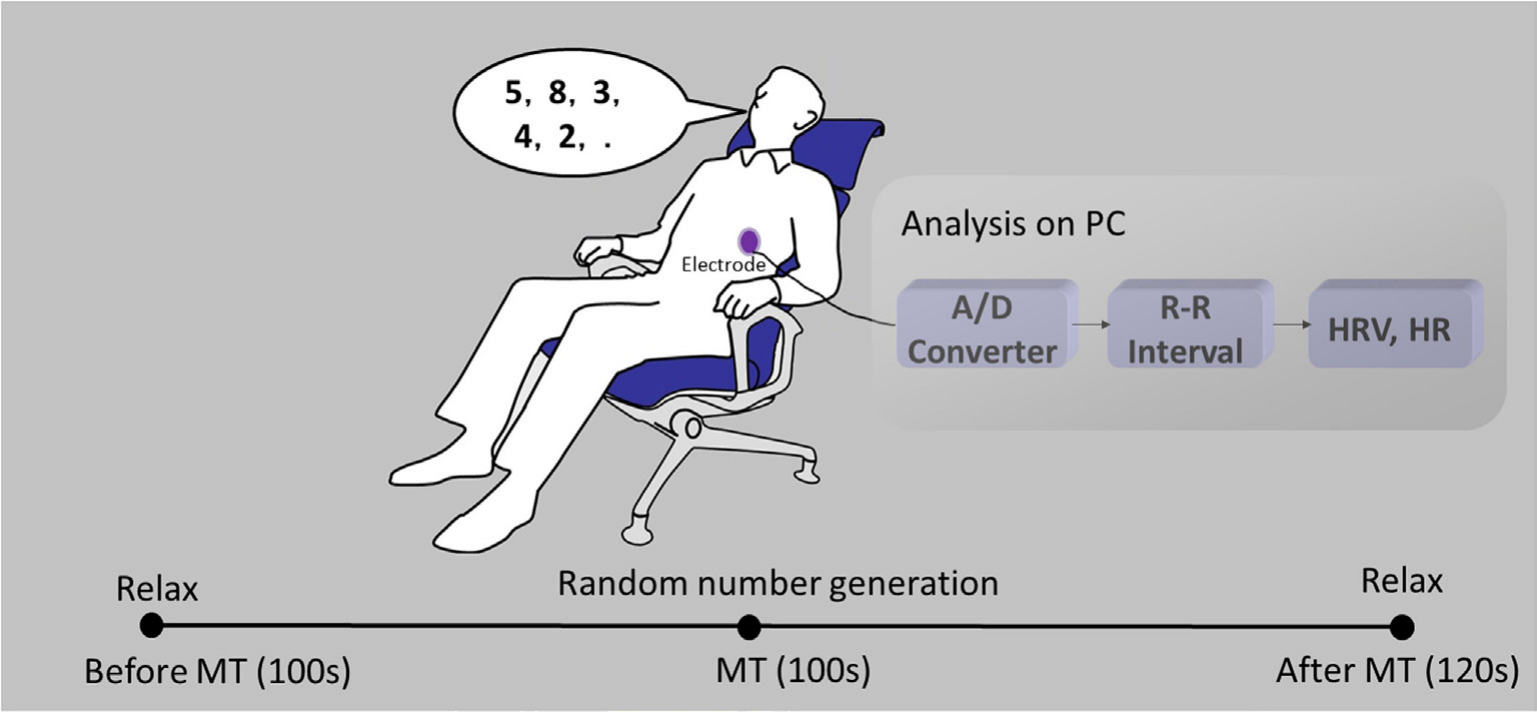
**A schematic diagram of heart rate variability (HRV) monitoring**. Electrocardiograms (ECGs) were recorded before, during, and after a mental task (MT).

The study protocol has previously been described (18). Briefly, each subject was seated in a chair with electrodes attached to their chest (Figure 1). Electrocardiograms (ECGs) were recorded before, during, and after the MT. Prior to the MT, subjects were instructed to relax for 100 s. Following this rest period, subjects completed random number generation for 100 s as the MT. During the MT, subjects were instructed to verbalize single-digit numbers (0–9) in a random order at a rate of 1 Hz with the assistance of a metronome. Following the MT, subjects were instructed to relax for 120 s. ECG signals were transferred to a computer *via* an A/D converter for offline analysis. Heartbeat intervals and HR were derived from R peak intervals. Power spectra of the time series of the heartbeat intervals were detected every 2 s for the preceding 30 s data using a maximum entropy method with the MemCalc software (GMS, Tokyo, Japan). To avoid the influence of preceding states, HF, LF/HF, and HR were averaged in the interim from 30 to 60 s after the onset of each new state.

### LRA Development Using Multivariate Data

Logistic regression analysis, which is a probabilistic statistical classification model, is a simple but powerful linear algorithm for analyzing multidimensional data and generating predictions of clinical outcomes. Multivariate selection is an important step in the development of a multivariate model that can effectively separate patients with MDD from the healthy control subjects. In the present study, we included nine HRV and HR indices in the LRA: HF_beforeMT_, HF_MT_, HF_afterMT_, LF/HF_beforeMT_, LF/HF_MT_, LF/HF_afterMT_, HR_beforeMT_, HR_MT_, and HR_afterMT_.

We defined the above selected nine variables as *x*_1_−*x*_9_ in a nine-dimensional vector *X* = (*x*_1_, *x*_2_, …, *x*_9_). LRA is a linear discriminative approach that attempts to optimize a logistic sigmoid function to calculate the probability *p* that vector *X* is representative of patients with MDD. The present study used binary classification; therefore, the probability of healthy control subjects is 1 − *p*. Thus, the LRA is expressed as follows:

(1) $$\text{log}\left(\frac{p}{1-p}\right)=b_{0}+b_{1}x_{1}+b_{2}x_{2}+\ldots +b_{9}x_{9}$$

where $\text{log}\left(\frac{p}{1-p}\right)$ is the predicted logit score, *b*_0_ is a constant, and *b*_1_, *b*_2_, …, *b*_9_ are regression coefficients estimated by maximum likelihood criterion. Probability *p* is written as the logistic sigmoid function:

(2) $$p=\frac{1}{1+e^{-(b_{0}+b_{1}x_{1}+b_{2}x_{2}+\cdots +b_{9}x_{9})}}$$

### Statistical Analysis

We evaluated the ability of the LRA to discriminate patients with MDD by calculating the sensitivity, specificity, positive predictive value (PPV), and negative predictive value (NPV). The performance of the LRA was evaluated using Nagelkerke’s *R*^2^ and Cox and Snell’s *R*^2^ tests (21). Moreover, to find the optimal cutoff point with the highest sensitivity and specificity of LRA, a receiver operating characteristic (ROC) curve was adopted. The area under the curve (AUC) of the ROC is considered an effective measure of the performance of a diagnostic test, with results ranging from 0.5 to 1.0, where larger values are indicative of better performance (22). The HRV and HR indices were compared between the classified MDD group and the healthy group *via* Student’s *t*-test. A *p*-value of less than 0.05 was considered to indicate statistical significance. The LRA and ROC were performed using SPSS 23.0 (IBM, Armonk, NY, USA).

## Results

**Figure 2:**
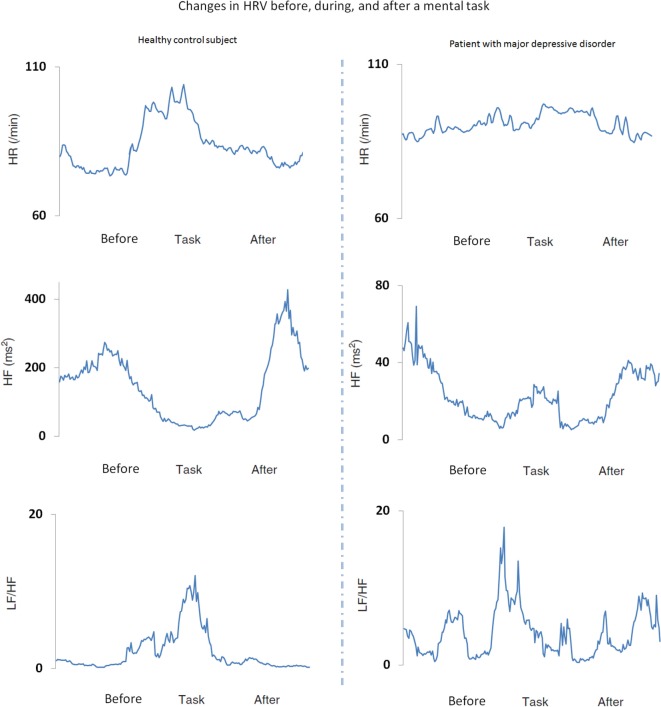
**Heart rate variability (HRV) and heart rate (HR) indices before, during, and after a mental task (MT) in a representative healthy control subject (left panels) and a patient with major depressive disorder (MDD) (right panels)**. The low frequency to high frequency ratio (LF/HF) (sympathetic index) and HR increased and HF (parasympathetic index) decreased during the MT condition in healthy control subjects, but not in patients with MDD.

Sample data from a patient with MDD and a healthy control subject are shown in Figure 2. In healthy control subjects, the LF/HF and HR increase, and HF decreases during the MT condition and return to baseline levels following the MT. In contrast, these changes are not observed in patients with MDD.

To discriminate patients with MDD from healthy control subjects in our dataset, the logistic sigmoid function $\text{log}\left(\frac{p}{1-p}\right)$ was calculated from the HRV and HR variables with the following estimated regression coefficients:

(3) $$\begin{array}{ll} \text{log}\left(\frac{p}{1-p}\right)= & -\;5.062+0.201{\text{HR}}_{\text{beforeMT}}-0.053{\text{HR}}_{\text{MT}}\\ & -0.086{\text{HR}}_{\text{afterMT}}\;-\;0.019{\text{HF}}_{\text{beforeMT}}+0.012{\text{HF}}_{\text{MT}}\\ & +0.007{\text{HF}}_{\text{afterMT}}+\;0.094\text{LF}/{\text{HF}}_{\text{beforeMT}}\\ & -0.087\text{LF}/{\text{HF}}_{\text{MT}}+0.312\text{LF}/{\text{HF}}_{\text{afterMT}} \end{array}$$

**Figure 3:**
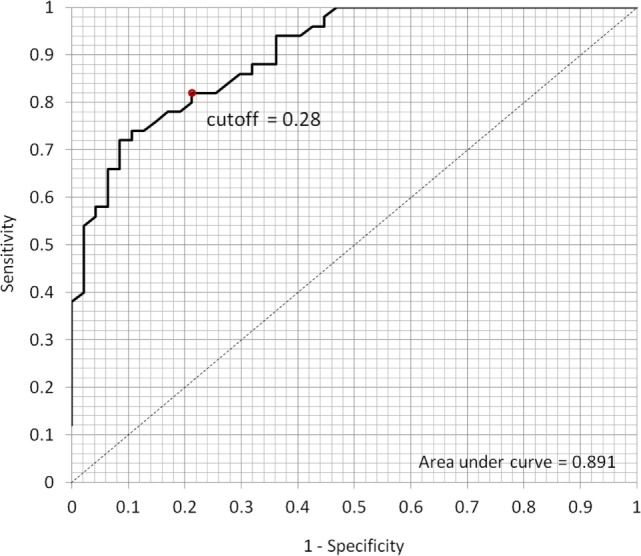
**Receiver operating characteristic (ROC) curve to determine the optimum cutoff point for classifying the patients with MDD and healthy subjects**. According to the ROC, the area under the curve for this screening test was 0.891 (95% confidence interval, 0.828–0.954); the sensitivity of 80.0% and specificity of 79.0% were obtained from the optimum cutoff point of 0.28.

**Figure 4:**
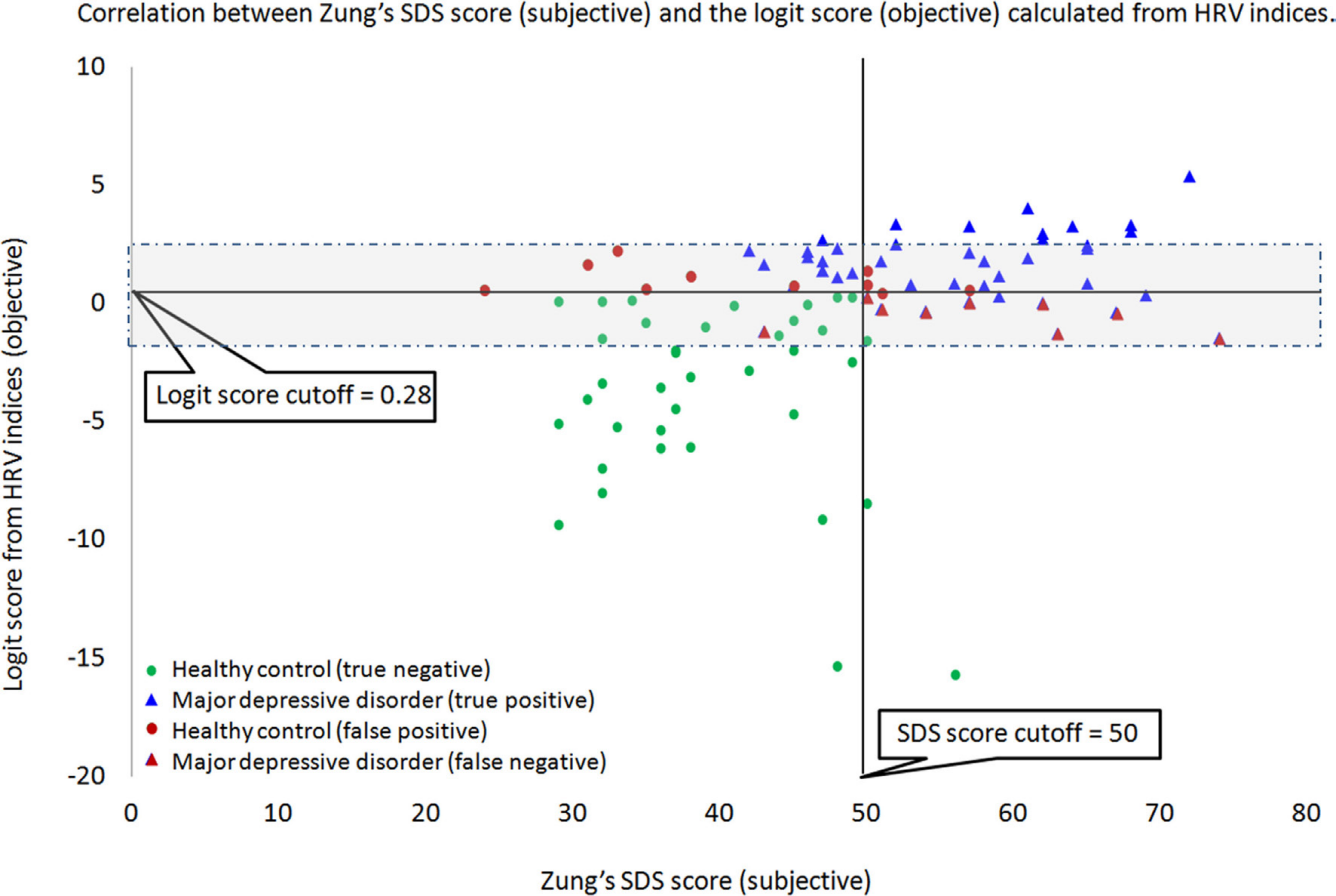
**With the cutoff point of 0.28, a total of 35 patients with major depressive disorder (MDD) (blue triangles) were predicted as patients with MDD by the logistic regression analysis (LRA); 9 patients (red triangles) with MDD were misdiagnosed as healthy**. Similarly, a total of 37 healthy control subjects (green circles) were predicted as healthy control subjects; 10 healthy control subjects (red circles) were misdiagnosed as patients with MDD. The gray area highlights the boundary region between patients with MDD and healthy control subjects, which demonstrates that the 9 false negatives and 10 false positives occur at the boundary region.

**Figure 5:**
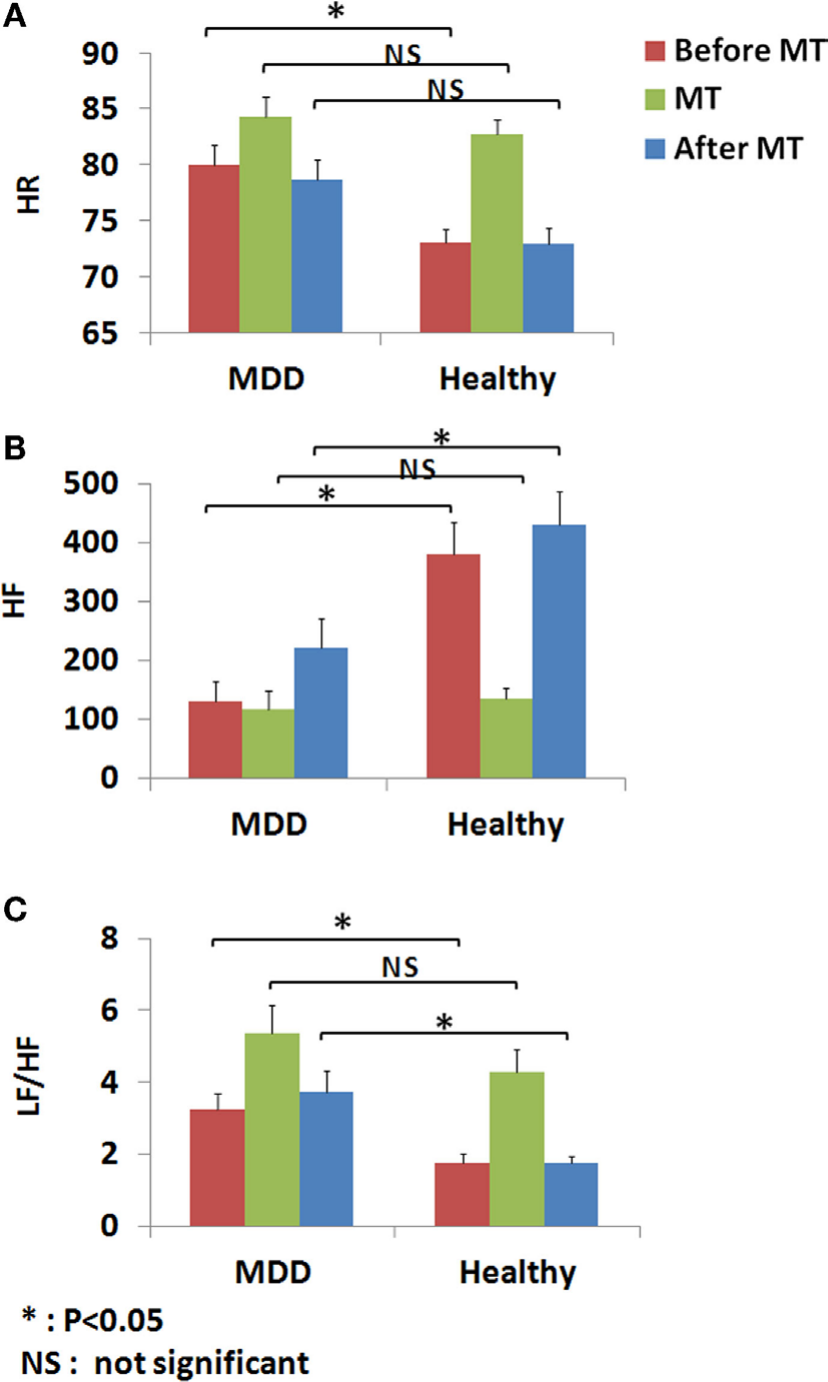
**Heart rate variability (HRV) and heart rate (HR) indices before, during, and after a mental task (MT) were compared within the MDD and healthy groups**. **(A)** HR of before MT differed significantly among MDD and healthy groups (*p* < 0.05), whereas no significant difference are found on MT and after MT conditions. **(B)** HF of before MT and after MT conditions differed significantly among MDD and healthy groups (*p* < 0.05), but no difference was present on MT conditions. **(C)** LF/HF of before MT and after MT conditions differed significantly among MDD and healthy groups (*p* < 0.05), but no difference on MT conditions.

The derived logistic sigmoid function $\text{log}\left(\frac{p}{1-p}\right)$ was statistically significant (*p* < 0.001). The Nagelkerke’s *R*^2^ and Cox and Snell’s *R*^2^ values were 0.614 and 0.461, respectively, which indicate that the derived logistic sigmoid function was useful in discriminating patients with MDD. The ROC analysis was performed to find the optimal cutoff point of predicted LRA logit score to discriminate the patients with MDD from healthy subjects (Figure 3). According to the ROC, the AUC for this screening test was 0.891 (95% confidence interval, 0.828–0.954), which indicated that the LRA was able to significantly distinguish between patients with MDD and the healthy group. In addition, a sensitivity of 80.0% and specificity of 79.0% were obtained from an optimum cutoff point of LRA logit score (0.28). Furthermore, a scatter plot was used to compare the results of our HRV-based objective screening method with subjective SDS scores (Figure 4). With this cutoff point, a total of 35 patients with MDD were predicted as patients with MDD by the LRA; 9 patients with MDD were misdiagnosed as healthy. Similarly, a total of 37 healthy control subjects were predicted as healthy control subjects; 10 healthy control subjects were misdiagnosed as patients with MDD. The corresponding PPV and NPV of the LRA were 78.0 and 80.4%, respectively. The gray area highlights the boundary region between patients with MDD and healthy control subjects, which demonstrates that the 9 false negatives and 10 false positives occur at the boundary region. In contrast, the sensitivity, specificity, PPV, and NPV were 72.0, 85.1, 82.0, and 76.9%, respectively, for the SDS score using a cutoff of 50. Thus, our HRV-based objective method using LRA significantly improved sensitivity and NPV compared with the SDS score alone. Logit scores also correlated significantly with subjective SDS score (*r* = 0.4, *p* < 0.05). Moreover, the HRV and HR indices were compared with the MDD and healthy groups. Figure 5A shows that the HR of before MT differed significantly among MDD and healthy groups (*p* < 0.05), whereas no significant difference are found on MT and after MT conditions. Figure 5B shows that the HF of before MT and after MT conditions differed significantly among MDD and healthy groups (*p* < 0.05), but no difference was present on MT conditions. Figure 5C shows that the LF/HF of before MT and after MT conditions differed significantly among MDD and healthy groups (*p* < 0.05), but no difference on MT conditions. Table 1 summarizes a more detailed comparison between our HRV-based objective method and patient-reported subjective SDS scores in part. In order to better understand the data, we sorted the table in descending order on logit scores. We found that the misclassified individuals gathered at close to the cutoff on both the patient-reported subjective SDS scores and HRV-based objective logit scores (gray areas). Moreover, the HRV-based screening method reduced both false negative and false positive error rate, comparing to the SDS method.

**Table 1:** **Objective logit scores based on heart rate variability (HRV) compared with patient-reported subjective self-rating depression scale (SDS) scores**.

Diagnosis	Objective screening (logit score ≥0.28)	Subjective screening (SDS score ≥50)	Age (years)	Sex
MDD	2.7	62	40	Male
MDD	2.2	42 (false negative)	50	Male
MDD	1.7	51	40	Male
MDD	1.3	47 (false negative)	24	Male
MDD	0.8	56	49	Male
MDD	0.8	65	51	Male
MDD	0.7	58	59	Female
MDD	0.3	59	52	Female
MDD	−1.1 (false negative)	43 (false negative)	65	Female
Healthy	0.7 (false positive)	50 (false positive)	33	Female
Healthy	0.5 (false positive)	57 (false positive)	32	Female
Healthy	0.4 (false positive)	51 (false positive)	35	Female
Healthy	0.2	48	21	Male
Healthy	−3.1	38	40	Male
Healthy	−3.3	32	27	Female
Healthy	−4.6	45	31	Female
Healthy	−5.2	33	30	Female
Healthy	−8.4	50 (false positive)	30	Female
Healthy	−15.3	48	25	Female
Healthy	−15.6	56 (false positive)	40	Female

## Discussion

We proposed an objective, non-invasive, HRV-based screening method for clinical risk stratification of patients with MDD using LRA. This method performed better than a subjective patient-reported screening method to discriminate patients with MDD. Existing diagnosis methods rely solely on the psychiatric clinician’s impression of the clinical presentation, which has minimal objectivity (23). A recent review reported that the accuracy of patient-reported and symptom-based diagnosis of MDD was approximately 47% (24). In this study, we obtained a similar result using patient-reported subjective SDS scores only. In contrast, our HRV-based objective method significantly improved screening accuracy.

Heart rate variability, which is a biomarker that reflects alterations in autonomic reactivity, is related to disturbed arousal function and can be used as a diagnostic measure and in clinical evaluation. In our previous studies, individual HRV and HR were shown to vary between depressed and non-depressed subjects (17, 18). However, there were significant data overlap between the two groups. The need to differentiate between patients with MDD and healthy control subjects led to the present study, which combined information from individual HRV and HR variables in LRA to improve screening accuracy in an automated way. The logit score output of this LRA can help psychiatric clinicians to make better diagnostic judgments. The logit score of the LRA can also be used to evaluate the severity of depression, and thereby aid the clinical risk stratification of patients with MDD.

The main limitation of this study was that our HRV dataset contains a total of 91 subjects, which is small compared to typical datasets from the field of classification of medical data and information. This is a limiting factor to the accuracy of our proposed MDD screening method. In statistical viewpoint, the dichotomization may result in the lost of information, so the performance to detect the relation between the variables and output will also be limited (25). However, in this study from the clinical viewpoint, dichotomization may be preferred because which offers a simple risk classification in setting screening criteria. In order to address these limitations, we plan to extend the current study to include a larger sample of clinical data for creating a more accurate logistic regression-based discriminant function. It is also important in the future study to apply the regression coefficients in the LRA on the present dataset to the data from newly recruited control subjects and patients with depression in order to consolidate the findings.

In addition, the MT paradigm is a provocative procedure used to differentiate between MDD and healthy subjects. The subject generates his or her own numbers in a random way for 100 s. This subject-generated method is a limitation that might create unintended subject-to-subject variability based on individual motivation, cooperation, and perhaps even current mood state. Therefore, further research should be done to investigate the subject-to-subject variation in this MT and apply other biomarkers, such as functional near-infrared spectroscopy, to measure the brain activity during subject generating random numbers. Moreover, in this study, we measured HRV and HR indices *via* ECG that requires electrodes to be attached to patients and can cause discomfort. We will expand this screening method to incorporate less invasive methods for HRV measurement, such as photoplethysmogram, microwave radar, or smart phone monitoring (26–28). These bio-measurement technologies, which can be combined with our proposed screening method without the need for electrode placement, allow for fully unconstrained HRV measurements to rapidly screen patients with MDD.

## Conclusion

In summary, the objective, non-invasive, HRV-based screening method developed in this study is effective in discriminating patients with MDD, which enables a higher screening sensitivity of 80.0% compared to the conventional subjective patient-reported screening method. Overall, the proposed method, which is based on a comprehensive analysis of HRV indices, has high potential for use as a novel objective tool in screening patients with MDD.

## Author Contributions

Study concept and design: GS, TS, TM, and TK. Acquisition of data: TS. Analysis and interpretation of data: GS, TS, and TM. Drafting of the manuscript: GS, TS, and TM. All the authors gave final approval of the version to be submitted.

## Conflict of Interest Statement

The authors declare that the research was conducted in the absence of any commercial or financial relationships that could be construed as a potential conflict of interest.
